# Prepare to persist

**DOI:** 10.1007/s00709-024-02016-y

**Published:** 2024-12-20

**Authors:** Peter Nick

**Affiliations:** Joseph Gottlieb Kölreuter Institute for Plant Sciences, Karlsruhe Institute of Technology, Karlsruhe, Germany

Although it has evaporated from the political agenda, global climate change exists and persists, posing risks and challenges on food security. Without intense research into the mechanisms of resilience, we will not be able to adjust our agriculture to this task. Shifting to new regions not been cultivated hitherto, offers no real alternative, since the arable land is limited and even progressively reduced by the spread of deserts and cities. Breeding for more resilient crops has gained momentum, but introgression of resilience factors, for instance from Crop Wild Relatives, while maintaining the specificities and yields of elite varieties is far from trivial and also time-consuming. Would it be possible to draw on *individual* experience of plants, such that the response to a challenge becomes promoted by the experience of previous challenges? This is, basically, the idea of stress priming. First proposed as plant counterpart of inducible immunity (Conrath et al. 2002), priming has later also been transferred to abiotic stress resilience. Priming can be operationalized as altered response to a stress factor depending on preceding encounters with either the same or other stress factors. It, thus, describes a memory effect on plant stress signaling and adaptive responses. Two contributions to the current issue demonstrate, how this memory can be integrated to promote stress resilience of crop plants.

The contribution by Amoah and Adu-Gyamfi 2024) investigates the possibility to induce priming by the experience of a mild drought episode. In wheat, this had been found to induce hardening against drought (Selote and Khanna-Chopra 2010). The current work not only confirms drought priming for foxtail millet (*Setaria italica*), but also addresses the mechanisms behind this phenomenon. This C_4_ plant is already endowed with considerable resilience against drought and is currently advocated in several countries suffering from global warming as alternative to other cereal crops. For this reason, the FAO has announced 2023 as “Year of the Millets” (Food and Agriculture Organization of the United Nations 2023). The authors focussed on the sugar metabolism monitoring both the metabolites, the activity of the relevant enzymes, and the transcripts encoding metabolic enzymes, but also a key player of source-sink transport, SWEET6, either in naïve plants, or in plants that had undergone drought priming. In the absence of stress, those parameters did not reveal any conspicuous differences, meaning that the memory of a mild drought episode does not alter the sugar homeostasis per se. However, when these plants were exposed to severe drought stress, the naïve plants responded by boosting sugar metabolism and the underlying genetic network, probably in order to compensate water scarcity by lowering the osmotic potential. In contrast, the drought-acclimated plants rather maintained the resting levels of these sugar-related parameters, meaning that the acclimation leads to a more robust buffering, avoiding the expensive strategy of sugar-driven osmotic adjustment, safeguarding resources and minimizing collateral damage, such as photorespiration. The impact of this work for application is evident—by imposing a mild drought stress (which might be achieved by restrained irrigation)—crop plants can be proofed against subsequent more severe stresses. Conceptually, these findings lead to the question, how drought priming can induce such a robust homeostasis that can buffer challenges more efficiently as in naïve plants.

The review by Pandey et al. (2024) revisits ancient agricultural practices, where smoke is used to increase the vigour of seeds. Stimulation of germination by fire is a common phenomenon in many therophytic species that need to adjust to perturbations of the canopy, because this temporary niche allows them to complete their lifecycle in the absence of competitors. The agents behind smoke-induced germination were identified as so called karrikins (derived from the Australian Aborigines word for “smoke”) that can interfere with strigolactone signaling. Some of the molecular components for this hijacked signaling have already been identified and are described in this contribution. The main target of the review is, however, to explore the possible applications of smoke water, being nothing else than a saturated solution of karrikins. There is accumulating evidence for a priming effect of smoke water not only improving seed vigour and germination success, but also performance under abiotic stress and plant immunity. Since stimulation of germination by smoke is a central factor for weed competitiveness, smoke water can also be used to lure dormant weeds into germination and, thus, to cleanse the seed bank of a plot prior to sowing. The review shows exemplarily, how mechanistic knowledge on stress signaling can help to evaluate and develop traditional agricultural practices to improve resilience of crop plants, even with simple technological means that are also available in developing countries.

Both contributions bridge mechanistic insight with real-world applications. They also illustrate a conceptual point. While it is widely accepted that animals are able to learn from experience, memory effects in plants have mostly been neglected and considered exotic, marginal phenomena. However, considering that developmental flexibility represents the central strategy for plant survival under adverse conditions, it is not astonishing at all that, in plants as well, the response to stress will be shaped by the history of previous stress encounters. Resilience breeding has been a powerful strategy to improve crop resilience—it is based on *collective* changes in the gene pool of the population. Stress priming could turn out to become an equally powerful strategy—acting complementarily with breeding, because it targets the *individual* stress history of plants. Considering the challenges imposed by climate change upon agriculture, it would be wise to pursue any possible strategy for mitigation.
